# FOCAL EVENTRATION OF THE DIAPHRAGM MASQUERADING AS INTRATHORACIC MASS- “PARTIAL VOLUME EFFECT”

**DOI:** 10.4103/0970-2113.59590

**Published:** 2008

**Authors:** Vishak Acharya, Ashvini Kumar, Rameshchandra Sahoo, R Anand, K Jayrama Shetty

**Affiliations:** 1 Assoc Prof Dept of TB & Respiratory Diseases, Kasturba Medical College Hospital, Mangalore; 3 Professor & Head, Dept of TB & Respiratory Diseases, Kasturba Medical College Hospital, Mangalore; 2 Assistant Professor Dept of Radiodiagnosis, Kasturba Medical College Hospital, Mangalore; 4 Assistant Professor Dept of Radiodiagnosis, Kasturba Medical College Hospital, Mangalore

## CASE HISTORY

**Fig. 1:**
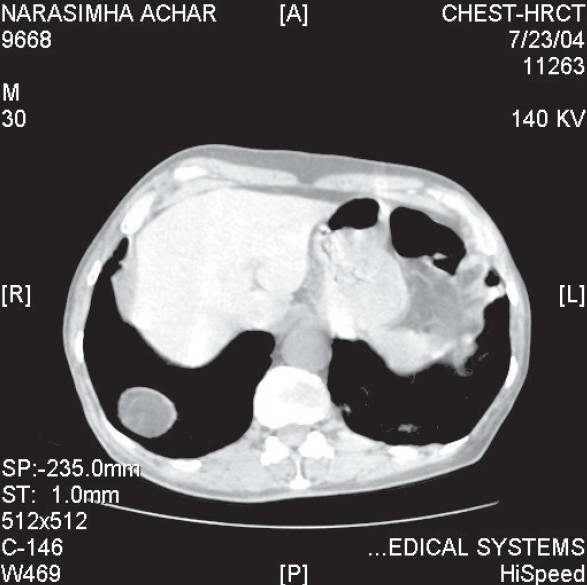
CT chest showing a right lower lobe intrapulmonary lesion of fat density.

**Fig. 2:**
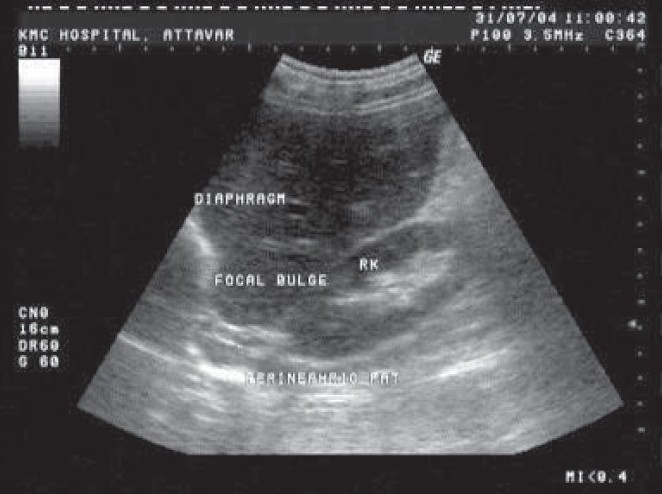
Ultrasonography showing an area of focal eventration of the diaphragm with adjacent protrusion of perinephric fat.

A 70 years old patient diagnosed to be suffering with COPD was being treated by us for an infective exacerbation. Frontal chest radiograph showed features of emphysema with hyperinflation. As it also showed right basal hypertransluceny and obscuring of the outline of the right diaphragm, a computerised tomogram of the chest was sought. CT chest revealed a well defined circular opacity in the right lower lobe (Fig-1). Densitometry study showed the of retroperitoneal fat was made. A CT Parasagittal reconstruction failed to show any focal defects in diaphragm, a possible cause for intrathoracic fat herniation. In view of this, the diagnosis was reviewed and a dynamic study in the form of ultrasonography of the chest was carried out (Fig-2). Ultrasonography chest showed a focal area of eventration of the diaphragm with restricted mobility.

## DISCUSSION

In CT imaging all structures represented in a slice are averaged and represented by a single CT number for unit surface of the image, known as pixel. Thicker the slice, the more averaging of adjacent structures occurs. This phenomenon is known as partial volume effect^1^. In areas of thorax in which tissue densities such as the mediastinum, chest wall, and the diaphragm do not demonstrate as much contrast as the lung parenchyma, partial volume effect may be observed^2^. The interpreter should be keenly aware of this problem in anatomic areas in which structural boundaries are running a near parallel or shallow oblique angle relative to the scanned plane. The partial volume effect may be detrimental in imaging of lower thorax, especially in the presence of a focal diaphragmatic abnormality like partial eventration which is quite common, as in our case. Partial volume effect may be overcome to certain extent by reducing the slice thickness by collimating the x-ray beam. This case also highlights the usefulness of dynamic imaging modality like ultrasonography, as adjunct to CT imaging to improve the diagnostic accuracy.
